# Synergistic Sensitization of Pancreatic Cancer Cells by Nanosecond Pulsed Electric Fields and Cold Atmospheric Plasma via Amplifying ROS and Apoptotic Signaling

**DOI:** 10.3390/ijms27135933

**Published:** 2026-07-01

**Authors:** Zobia Minhas, Edwin A. Oshin, Lifang Yang, Chunqi Jiang, Siqi Guo

**Affiliations:** 1 Frank Reidy Research Center for Bioelectrics, Old Dominion University, Norfolk, VA 23508, USA; zminh001@odu.edu (Z.M.); cjiang@odu.edu (C.J.); 2 Department of Electrical and Computer Engineering, Old Dominion University, Norfolk, VA 23529, USA; 3 Leroy T. Canoles Jr. Cancer Research Center, Department of Biomedical and Translational Sciences, Macon & Joan Brock Virginial Health Sciences, Old Dominion University, Norfolk, VA 23501, USA; yanglf@odu.edu

**Keywords:** nanosecond pulsed electric fields (nsPEF), cold atmospheric plasma (CAP), pancreatic cancer, synergy, reactive oxygen species (ROS), mitochondrial dysfunction, apoptosis

## Abstract

Pancreatic cancer remains a highly lethal malignancy, with standard therapies offering limited benefits in advanced stages; thus, novel strategies that exploit specific cancer cell vulnerabilities are urgently needed. Building on our previous findings that nanosecond pulsed electric fields (nsPEF) combined with cold atmospheric plasma (CAP) produce enhanced cytotoxicity, this study investigates the molecular mechanisms underlying this synergy. Pan02 pancreatic cancer cells were subjected to nsPEF, CAP, or a combination of both. We assessed cell viability, reactive oxygen species (ROS) production, and mitochondrial integrity using metabolic assays, flow cytometry, and fluorescence microscopy. Apoptotic markers were evaluated via Western blotting and caspase activity assays. Combined nsPEF–CAP treatment significantly outperformed either modality alone in inducing cell death. Mechanistically, dual treatment triggered a surge in intracellular ROS, particularly mitochondrial superoxide, indicating severe oxidative stress. Distinct mitochondrial responses were observed: nsPEF reduced mitochondrial membrane potential, whereas CAP alone caused a slight elevation. Notably, while CAP induced apoptosis (evidenced by increased cleaved caspase-3 and caspase-3/7 activity), lethal nsPEF (100 pulses) caused cell death without triggering apoptotic signaling. However, mild nsPEF (20 pulses) significantly potentiated CAP-induced apoptosis. These findings suggest that nsPEF sensitizes cells to CAP treatment by amplifying oxidative stress and mitochondrial dysfunction. This synergistic combination represents a promising therapeutic approach for managing pancreatic cancer cells resistant to conventional therapies.

## 1. Introduction

Despite decades of research, pancreatic cancer remains characterized by exceptional lethality and a profound intrinsic resistance to conventional chemotherapeutic and immunotherapeutic interventions [1,2,3]. This poor prognosis is largely attributed to the tumor’s dense stromal barrier, early metastatic dissemination, and limited response to chemotherapy [4] and radiation [5]. Survival statistics remain persistently low, with an overall 5-year survival rate of approximately 12% across all stages [6]. For patients diagnosed with distant metastatic disease, the prognosis is particularly dismal, with a 5-year survival rate of only ~2.9% [7]. Therefore, novel therapeutic strategies are urgently needed to overcome these barriers and improve patient outcomes.

Nanosecond pulsed electric field (nsPEF), also known as nano-pulse stimulation, has emerged as a promising non-thermal physical modality for cancer ablation [8,9,10]. nsPEF employs ultra-short, high-voltage pulses [11,12,13] to induce nanoporation in the plasma membrane and affect intracellular structures, including the mitochondria, endoplasmic reticulum, and nucleus [14,15,16,17]. These interactions disrupt calcium signaling, [11,18,19,20] and mitochondrial homeostasis [11], while generating reactive oxygen species (ROS), ultimately triggering both apoptotic and non-apoptotic cell death pathways. Beyond direct ablation, nsPEF can sensitize tumors to drug delivery, gene transfer, and immune activation [21,22]. While efficacy has been demonstrated in melanoma [23], breast [24], pancreatic [21], and hepatocellular carcinoma [8,25], recent studies highlight its potential to suppress tumor growth and reduce multi-drug resistance in pancreatic cancer models [26,27].

However, the therapeutic efficacy of nsPEF is currently constrained by tumor volume. At diagnosis, human pancreatic tumors typically exceed 2 cm, presenting a challenge for effective local nsPEF treatment. Preclinical studies indicate an inverse relationship between tumor size and nsPEF efficacy: while small tumors (4–7 mm) exhibit high regression rates, significantly reduced tumor control is observed in larger tumors (8–11 mm) treated with identical parameters [21]. This suggests that nsPEF monotherapy may be insufficient for durable control of clinically relevant pancreatic masses, highlighting the need for combinatorial strategies that enhance efficacy without escalating treatment intensity.

Cold atmospheric plasma (CAP) offers a complementary approach. CAP is a non-thermal plasma generated at atmospheric pressure [28] comprising a dynamic mixture of reactive oxygen and nitrogen species (ROS/RNS), UV photons, and charged particles [29,30,31]. The synergistic interplay of long-lived (e.g., H_2_O_2_, NO_2_^−^) and short-lived species (e.g., •OH, ^1^O_2_) [32,33,34] allows CAP to selectively inactivate cancer cells by exploiting their altered redox homeostasis [29]. CAP induces diverse cell death pathways, including apoptosis, ferroptosis [35], and pyroptosis [36], and has demonstrated potential in stimulating anti-tumor immunity [13,37,38,39]. Importantly, CAP-based technology has been developed for early clinical trials, where it was used to clear post-surgical tumor residuals [40]. Importantly, despite these advantages, CAP monotherapy faces significant limitations, primarily regarding tissue penetration. Therapeutic effects of plasma are typically restricted to superficial cell layers (3–5 cell layers) [41,42], making complete eradication of deep-seated or non-superficial lesions difficult [43,44]. Furthermore, the complexity of in vivo mechanisms and the lack of standardized “plasma dosing” complicates its independent clinical application [45,46].

The combination of nsPEF and CAP presents a compelling theoretical advantage: nsPEF acts as an intracellular stressor that permeabilizes membranes, potentially facilitating the entry of CAP-generated reactive species [31,47]. Building on prior work demonstrating the synergistic cytotoxicity of this combination in pancreatic cancer [31], the specific molecular mechanisms driving this effect remain undefined. While both modalities induce ROS, the extent to which nsPEF modulates intracellular and mitochondrial ROS to sensitize cells to CAP is unknown. To address this gap, this study investigates ROS generation, mitochondrial oxidative stress, and apoptotic signaling in pancreatic cancer cells treated with combined nsPEF and CAP. By specifically examining mild-to-sublethal nsPEF conditions, we aim to elucidate how nanosecond pulses alter cellular susceptibility to CAP-induced oxidative damage.

## 2. Results

### 2.1. Combined nsPEF–CAP Treatment Markedly Reduces Pancreatic Cancer Cell Viability

**Figure 1:**
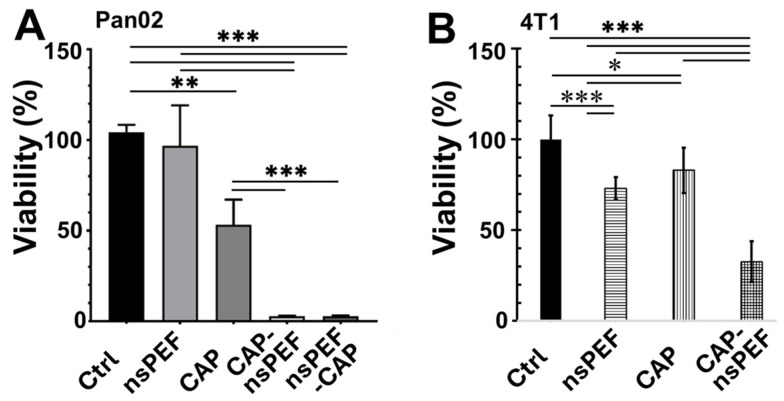
Effect of combined nanosecond pulsed electric field and cold atmospheric plasma treatment on cancer cell viability. Cancer cells were subjected to various treatments: untreated control (Ctrl), nanosecond pulsed electric field (nsPEF) alone (60 ns, 50 kV/cm, 1 Hz, and 20 pulses), cold atmospheric plasma alone with 3 min plasma treatment time (CAP), sequential application of CAP followed by nsPEF (CAP-nsPEF), or sequential application of nsPEF followed by CAP (nsPEF-CAP). Cell viability was assessed 24 h (h) post-treatment using a WST-1 metabolic activity assay. (**A**) Viability of Pan02 cells treated with Ctrl, nsPEF, CAP, CAP-nsPEF, and nsPEF-CAP. (**B**) Viability of 4T1 cells treated with Ctrl, nsPEF, CAP, and CAP-nsPEF. Data is presented as mean ± SD.*: *p* < 0.05, **: *p* < 0.01, ***: *p* < 0.001 (one-way ANOVA).

First, to confirm our previous finding of synergy between nsPEF and CAP [31], we treated Pan02 pancreatic cancer cells using mild-to-modest parameters. nsPEF parameters were set to a pulse duration of 60 ns, 50 kV/cm, 1 Hz, and 20 pulses. CAP was generated using an atmospheric pressure plasma jet with nanosecond high-voltage pulses (200 ns, 9 kV, 2 kHz) for a 3 min exposure. Cells were treated with either modality alone or in combination sequences (CAP → nsPEF or nsPEF → CAP), as shown in Figure 1A. Cell viability, assessed 24 h post-treatment using the WST-1 assay, revealed that nsPEF and CAP monotherapies reduced viability by 7.3% and 49.3%, respectively, relative to untreated controls. In contrast, the combined CAP → nsPEF and nsPEF → CAP treatments resulted in near-complete suppression of metabolic activity, causing 97.4% and 97.3% cell death, respectively. This profound cytotoxicity substantially exceeds the theoretical additive effect of the individual treatments, confirming a strong synergistic interaction that severely compromises cell survival [31].

This synergistic interaction between nsPEF and CAP was consistently observed in different cancer models. As shown in Figure 1B, the combination of CAP and nsPEF induced 67.4% cell death in 4T1 breast cancer, whereas nsPEF (35 pulses) or CAP (3 min) alone resulted in only modest cell death rates of 27.0% or 17.0%, respectively. To quantify this interaction, we calculated the synergistic quotient (SQ), defined as the ratio of the effect of combination treatment to the sum of two individual treatment effects. The calculated SQ was 1.87 for Pan02 cells and 1.53 for 4T1 cells. Since both SQs are greater than 1, these data confirm that the combination of nsPEF and CAP generates a true synergistic effect in both cancer cell lines tested.

Given that both nsPEF and CAP are known inducers of ROS [29,48,49], we investigated the relationship between ROS generation next, and this enhanced cell death.

### 2.2. nsPEF–CAP Treatments Elevate Mitochondrial Superoxide and Cytoplasmic ROS Production

**Figure 2:**
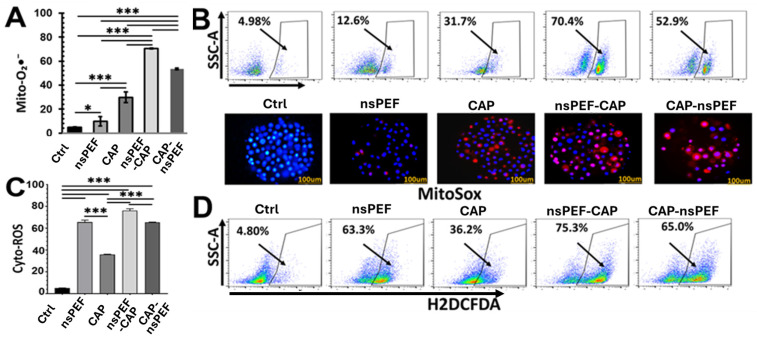
Effect of combined nanosecond pulsed electric field and cold atmospheric plasma treatment on reactive oxygen species generation in Pan02 cells. Pan02 cells were treated with nanosecond pulsed electric field (nsPEF, 60 ns, 50 kV/cm, 1 Hz, and 20 pulses) alone, cold atmospheric plasma (CAP, 3 min plasma treatment time) alone, sequential application of nsPEF followed by CAP (nsPEF-CAP), or sequential application of CAP followed by nsPEF (CAP-nsPEF). The control group (Ctrl) represents untreated cells maintained in media only. All assessments were performed at 2 h post-treatment. (**A**) Percentage of positive cells for mitochondrial superoxide (Mito-O_2_*^−^). (**B**) Representative flow plots and fluorescent microscopic images using MitoSOX (5 µM) staining (red) and DAPI nuclear staining (blue). (**C**) Percentage of positive cells for cytoplasmic ROS. (**D**) Representative flow plots using H2DCFDA staining. Data is presented as mean ± SD. *: *p* < 0.05 and ***: *p* < 0.001 (one-way ANOVA).

Flow cytometry analysis revealed distinct patterns of mitochondrial superoxide production across the treatment groups. While both nsPEF and CAP monotherapies increased MitoSOX fluorescence compared to the control, CAP induced significantly higher levels than nsPEF (Figure 2A). The percentages of cell populations exhibiting elevated mitochondrial superoxide were 4.6% (control), 9.9% (nsPEF), 29.8% (CAP), 70.6% (nsPEF → CAP), and 53.5% (CAP → nsPE). Notably, the highest levels of mitochondrial superoxide were detected in the combination groups. The nsPEF → CAP sequence, in particular, showed a marked amplification of ROS that far exceeded the additive effects of the individual treatments. SQ analysis yielded values of 1.78 for the nsPEF → CAP sequence and 1.35 for the CAP → nsPEF sequence. This synergistic elevation demonstrates that the integration of nsPEF and CAP, especially when nsPEF acts as the priming stimulus, intensifies mitochondrial oxidative stress more effectively than either modality alone.

To visually confirm these findings, we evaluated mitochondrial ROS using fluorescence microscopy. Control and single-treatment groups exhibited weak-to-moderate MitoSOX staining (Figure 2B), consistent with flow cytometry data (Figure 2A). In contrast, both combination treatments produced bright, widespread mitochondrial ROS signals, reinforcing that dual exposure drives robust superoxide accumulation at the mitochondrial level.

Cytoplasmic ROS levels were primarily influenced by nsPEF. nsPEF monotherapy triggered a strong increase in H_2_DCFDA fluorescence, whereas CAP produced only a moderate elevation. In the combination groups, nsPEF → CAP resulted in higher cytoplasmic ROS levels than either single treatment; however, the magnitude of this increase did not exceed the sum of the individual effects. The CAP → nsPEF sequence did not enhance cytoplasmic ROS beyond the nsPEF baseline (Figure 2C). Overall, no synergistic generation of general cytoplasmic ROS was observed, despite the combination treatments resulting in >97% cell death compared to only 7% for nsPEF alone. The discrepancy between cytoplasmic ROS levels and cell viability suggests distinct mechanisms are at play and will be addressed in the discussion. Representative flow cytometry plots (Figure 2D) confirmed these trends: nsPEF shifted the population markedly to higher ROS levels, while CAP produced a smaller shift, and the combination showed a further but non-additive increase [50,51,52,53].

### 2.3. ROS Scavenging Rescues Cells from Dual Treatment-Induced Death

**Figure 3:**
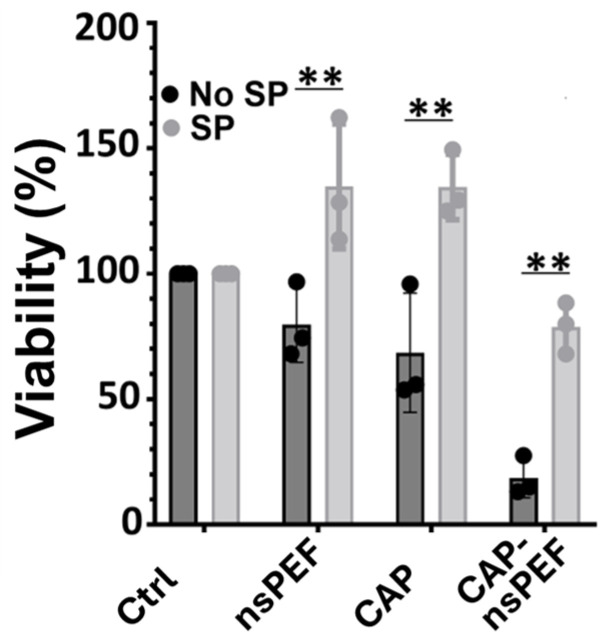
Protection of Pan02 cells from ROS-induced death by sodium pyruvate. Pan02 cells were subjected to various treatments: nanosecond pulsed electric field (nsPEF, 60 ns, 50 kV/cm, 1 Hz, and 20 pulses) alone, cold atmospheric plasma (CAP, 3 min plasma treatment time) alone, or sequential application of CAP followed by nsPEF (CAP-nsPEF). Untreated cells served as controls (Ctrl). Prior to treatment, cells were pre-incubated with sodium pyruvate (SP) or without (No SP). Cell viability was assessed 24 h post-treatment using a WST-1 metabolic activity assay, with results expressed as a percentage of untreated control cells (Ctrl). Data is presented as mean ± SD., **: *p* < 0.01 (two-way ANOVA).

To determine whether ROS are the key drivers of cytotoxicity, we evaluated cell viability in the presence of the antioxidant sodium pyruvate. Cells were pre-incubated with sodium pyruvate (10 mM) for 30 min prior to treatment, and viability was quantified 24 h later (Figure 3). In the absence of an antioxidant, nsPEF alone maintained 79.8% viability, while CAP reduced viability to 68.5%. As observed previously, the combined CAP → nsPEF treatment caused a profound loss of metabolic activity, dropping viability to 18.6%. Importantly, sodium pyruvate alone did not increase viability in untreated controls. However, in the presence of sodium pyruvate, the cytotoxic effects were completely reversed. Interestingly, both nsPEF and CAP single treatments in the presence of the scavenger resulted in metabolic activity exceeding 100% (nsPEF + SP: 134.8%; CAP + SP: 134.6%). Although the exact mechanism underlying this unexpected increase in metabolic activity remains unclear, potential contributions from partial ROS quenching and/or treatment-induced alterations in mitochondrial membrane potential (ΔΨm), including mitochondrial hyperpolarization, should be considered. Crucially, sodium pyruvate rescued the combination group, restoring viability from 18.6% to 78.8%. These findings demonstrate that ROS generation is an essential driver of cell death following both single and combined exposures. The strong protective effect of sodium pyruvate highlights oxidative stress as the central contributor to the synergistic cytotoxicity.

### 2.4. nsPEF Induces Mitochondrial Depolarization Whereas CAP Hyperpolarizes Mitochondria

**Figure 4:**
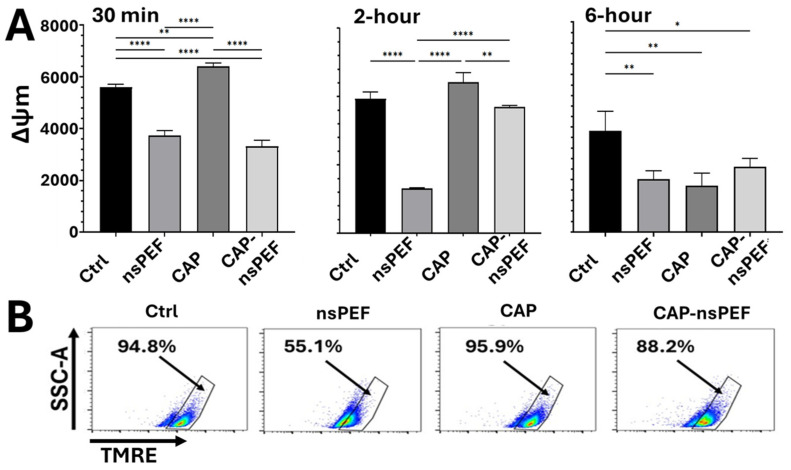
Distinct impact of nanosecond pulsed electric field and cold atmospheric plasma on mitochondrial membrane potential ΔΨm in Pan02 cells. Pan02 cells were treated with media control (Ctrl), nanosecond pulsed electric field (nsPEF, 60 ns, 50 kV/cm, 1 Hz, and 20 pulses) alone, cold atmospheric plasma (CAP, 3 min plasma treatment time) alone, and sequential application of CAP followed by nsPEF (CAP-nsPEF). ΔΨm was assessed at 30 min, 2 h, or 6 h post-treatment using the TMRE fluorescent probe. (**A**) Bar graph shows ΔΨm, indicated by TMRE fluorescence intensity. (**B**) Representative flow cytometric plots illustrating ΔΨm shift at the 2 h time point. Data is presented as mean ± SD. * *p* < 0.05, ** *p* < 0.01, **** *p* < 0.0001 (one-way ANOVA).

To investigate mitochondrial contributions to cell death, we measured the mitochondrial membrane potential (ΔΨm) using TMRE at 30 min (min), 2 h, and 6 h post-treatment and compared these early changes to 24 h viability data. nsPEF alone induced a significant decrease in ΔΨm lasting up to 6 h, reducing TMRE intensity to approximately two-thirds of the control baseline (Figure 4A). However, despite this depolarization, cell viability remained largely unaffected (only 7.3% reduction, Figure 1), suggesting that this level of ΔΨm loss is likely mild, transient, or reversible, and not independently lethal.

In contrast, CAP treatment for 3 min, which caused ~50% cell death (Figure 1), resulted in a modest increase (hyperpolarization) in ΔΨm from 30 min to 2 h but a significant decrease (depolarization) at 6 h compared to controls (Figure 4A,B). This indicates a lack of direct correlation at early time points between depolarization and CAP-induced cell death. Combination treatments (CAP → nsPEF) resulted in near-complete cell death (>97%), yet the observed ΔΨm showed inconsistent changes with either the same level reduction as nsPEF treatment at 30 min and 6 h or a mild reduction at 2 h (for CAP → nsPEF) (Figure 4A,B). Therefore, the dramatic enhancement of cytotoxicity in combination treatments cannot be attributed primarily to gross alterations in ΔΨm. While ΔΨm changes can correlate with cell death under some conditions [15], in this system, early ΔΨm shifts were not predictive of long-term survival.

### 2.5. Enhanced Apoptosis Likely Contributes to Synergistic Cytotoxicity of nsPEF-CAP Combination Treatment

**Figure 5:**
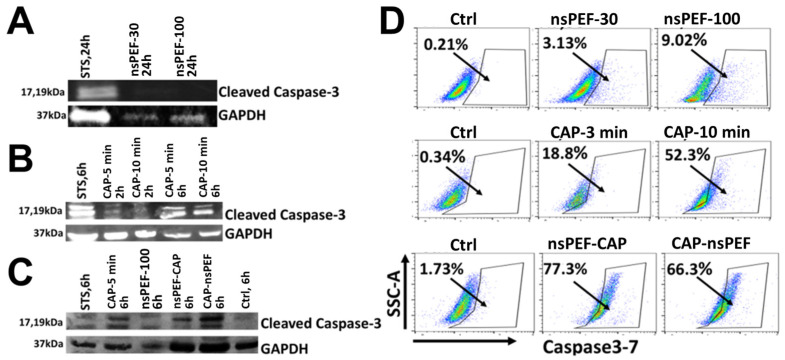
The analysis of caspase-3 cleavage and caspase-3/7 activity in Pan02 cells following nanosecond pulsed electric field and cold atmospheric plasma treatments. Pan02 cells were subjected to various treatments with nanosecond pulsed electric field (nsPEF; 60 ns, 50 kV/cm, and 1 Hz) and/or cold atmospheric plasma (CAP). Staurosporine (STS) served as a positive control for apoptosis induction. Representative image of Western blot for cleaved caspase-3 expression in Pan02 cells treated with nsPEF 30 pulses or 100 pulses at 24 h (**A**); CAP treatment 5 or 10 min at 2 or 6 h (**B**); and nsPEF 100 pulses, CAP-5 min, nsPEF 20 pulses followed by CAP-3 min (nsPEF-CAP), and CAP-3 min followed by nsPEF 20 pulses (CAP-nsPEF) at 6 h (**C**). (**D**) Representative flow cytometric plots illustrating caspase 3/7 activity in Pan02 cells 24 h post-treatment. Cells were untreated or treated with nsPEF (30 pulses or 100 pulses), CAP (3 min or 10 min), or sequential combined treatments (nsPEF-CAP: nsPEF 20 pulses followed by CAP-3 min; CAP-nsPEF: CAP-3 min followed by nsPEF 20 pulses).

To determine the mode of cell death, we first investigated whether nsPEF induces apoptosis. Pan02 cells were treated with sublethal or lethal doses of nsPEF (30 or 100 pulses), resulting in 20% or >80% cell death, respectively [31]. As shown in Figure 5A, cleaved caspase-3 was detected only in the positive control (staurosporine); no cleavage was observed in nsPEF-treated cells. This demonstrates that, under the parameters tested, nsPEF induces non-apoptotic cell death in Pan02 cells.

Next, we assessed CAP-induced apoptosis. Cells treated with CAP (5 or 10 min) showed clear, time-dependent caspase-3 cleavage (17 kDa and 19 kDa fragments), with stronger activation observed at 6 h post-treatment compared to 2 h (Figure 5B). This confirms that CAP robustly activates apoptotic pathways at the protein level.

Having established that CAP engages apoptotic signaling while nsPEF does not, we examined the combination. As shown in Figure 5C, both sequential treatments significantly enhanced caspase-3 cleavage compared to either modality alone. To quantify this, we employed a functional caspase-3/7 activity assay. As shown in Figure 5D, nsPEF alone induced minimal caspase activity (3.13% positive cells for 30 pulses; 9.02% for 100 pulses). In contrast, CAP treatment markedly enhanced apoptosis in a dose-dependent manner (18.8% positive for CAP 3 min; 52.3% positive for CAP 10 min). Notably, the combination of mild nsPEF (20 pulses) followed by CAP (3 min) resulted in the strongest caspase-3/7 activation (77.3% positive cells), while CAP followed by nsPEF yielded 66.3% positive cells. These results suggest that although nsPEF does not inherently trigger apoptosis, it effectively primes cells to undergo CAP-induced apoptosis. Collectively, these data demonstrate that nsPEF functions as a sensitizer, synergistically amplifying the caspase-dependent apoptotic cascade initiated by CAP.

## 3. Discussion

In this study, we demonstrate that nanosecond pulsed electric fields (nsPEF) and cold atmospheric plasma (CAP) act synergistically to induce cell death in both Pan02 pancreatic and 4T1 breast cancer models, with the combined treatment yielding substantially greater cytotoxicity than either modality alone. Our findings suggest that this synergy arises from nsPEF sensitizing cells, via ROS amplification, to CAP-induced apoptosis. Mechanistically, we show that while CAP is the primary driver of ROS-mediated apoptosis, nsPEF functions as a “primer” that escalates this process. Notably, lethal nsPEF alone failed to activate apoptotic signaling, instead inducing non-apoptotic cell death. Furthermore, we observed a distinct divergence in mitochondrial responses: changes in mitochondrial membrane potential (ΔΨm) did not correlate with cell death levels across treatments, challenging the assumption that early depolarization is a prerequisite for cytotoxicity in this context [54]. A common paradigm posits that extracellular CAP-generated ROS penetrate the cell or stimulate endogenous ROS production, subsequently activating downstream death pathways [55,56]. Indeed, recent reports have linked CAP-induced ROS to alternative death mechanisms, such as GSDME-dependent pyroptosis [36]. Beyond ROS, CAP alters membrane permeability and ion flux, particularly calcium homeostasis [57,58]. Mild increases in intracellular Ca^2+^ can stimulate mitochondrial dehydrogenases in the TCA cycle and transiently enhance electron transport chain (ETC) activity, resulting in elevated ΔΨm [39]. Such hyperpolarization likely reflects an early stress-adaptation response, where cells temporarily boost bioenergetic output to counteract oxidative stress. Consistent with this “biphasic” model, we observed that ΔΨm remained intact or even hyperpolarized at 2 h post-treatment, despite robust ROS accumulation. However, by 6 h, prominent caspase-3 activation occurred, indicating that sustained oxidative stress eventually overwhelmed this adaptive capacity, triggering mitochondrial dysfunction and apoptosis. The complete rescue of cell viability by antioxidants confirms that ROS are the indispensable mediators of this CAP-induced toxicity [38,39,59,60].

In stark contrast, nsPEF alone produced significant early ROS and disrupted ΔΨm but failed to activate caspase-3, even under conditions causing lethal (>90%) cytotoxicity. This absence of caspase cleavage indicates that nsPEF predominantly triggers non-apoptotic cell death in Pan02 cells. This finding suggests that early mitochondrial depolarization alone is insufficient to trigger robust apoptotic signaling in Pan02 cells. While nsPEF caused the greatest reduction in ΔΨm at both 30 min and 2 h, it generated substantially less caspase-3 activation than CAP treatments. Conversely, CAP initially induced mild mitochondrial hyperpolarization before progressing to marked depolarization at later time points, despite being the stronger apoptotic inducer. These observations indicate that the magnitude of early ΔΨm disruption does not directly predict apoptotic outcome. Instead, sustained oxidative stress and downstream apoptotic signaling are more closely associated with treatment-induced cell death, aligning with observations by Pakhomova et al. regarding delayed or absent apoptotic features in certain cell lines [61]. Collectively, these data suggest that classical apoptosis contributes marginally to nsPEF monotherapy efficacy. Crucially, however, mild nsPEF, which was non-lethal on its own, markedly enhanced CAP-mediated apoptosis in the combination setting. This supports a model where nsPEF functions as a priming stimulus, increasing cellular susceptibility to the ROS overload delivered by CAP. Our results also reveal an intriguing divergence in mitochondrial behavior. Time-course analysis demonstrated that nsPEF caused the greatest early drop in ΔΨm at both 30 min and 2 h [15,62,63,64], whereas CAP induced transient mitochondrial hyperpolarization during the early phase before progressing to marked depolarization by 6 h, despite being the stronger apoptotic inducer later. This mismatch suggests: (1) early ΔΨm depolarization is neither the initial cause of CAP-induced apoptosis nor the primary driver of synergistic cell death; and (2) severe ΔΨm loss in CAP-treated cells likely occurs as a secondary consequence of downstream caspase execution [65,66]. While previous studies in melanoma cells have linked nsPEF-induced depolarization directly to cell death, the dissociation observed here suggests that in Pan02 cells, the nsPEF-induced potential loss is likely transient or reversible [15] and not the sole executioner mechanism.

Although we clearly demonstrate that combination treatment amplifies ROS and cytotoxicity, several specific interactions warrant further study. The sources of ROS differ fundamentally: CAP delivers extracellular species (e.g., H_2_O_2_, NOx) entering via diffusion or peroxiporins [44], while nsPEF induces intracellular ROS via mitochondrial and calcium-dependent pathways [49]. The synergy likely stems from nsPEF-induced nanoporation facilitating the influx of CAP-derived species, while simultaneous calcium mobilization compounds mitochondrial stress. Prior studies indicate that such sustained oxidative stress can activate intrinsic apoptotic [11], providing a plausible framework for the enhanced caspase-3/7 activity we observed.

Integrating these observations, we propose a synergistic model where CAP and nsPEF operate via complementary, temporally distinct mechanisms converging on catastrophic oxidative stress. CAP provides a rich source of extracellular RONS, which enter cells, potentially facilitated by nsPEF-induced nanopores [65,67] while nsPEF simultaneously triggers endogenous ROS production and calcium influx. This “double hit” overwhelms antioxidant defenses and the endoplasmic reticulum [49,68,69], driving the cell past a survivable threshold. Notably, the lack of caspase activation by lethal nsPEF alone confirms its role is not to initiate apoptosis directly, but to create a pro-oxidant state that amplifies the efficacy of CAP, which acts as the apoptotic inducer [70,71,72].

Several limitations of this study should be acknowledged. First, while we have established a clear link between the intracellular ROS burst and cell demise, the precise molecular intermediates connecting oxidative stress to the activation of specific caspases remain to be fully mapped. Although our data point toward apoptosis, the potential involvement of other regulated cell death forms, such as ferroptosis, pyroptosis, or necroptosis, under varying pulse parameters requires further investigation. Second, this study was conducted in vitro using two murine cancer cell lines (Pan02 and 4T1). Because in vivo tumors are characterized by dense stroma, complex vasculature, and a dynamic tumor microenvironment, these factors may influence the delivery and efficacy of nsPEF and CAP. Future research should prioritize validation in 3D organoid systems and immuno-competent animal models to evaluate the translational potential and systemic immune implications of this combinatorial approach.

Although the nsPEF–CAP combination remains at an early stage of development and has thus far been evaluated only in vitro, the observed synergistic effect suggests several potential translational applications that warrant future investigation in animal models. First, this approach may enhance the elimination of residual tumor cells at surgical margins. CAP has been explored clinically as a local treatment for advanced solid tumors and for reducing residual disease following tumor resection [73]. The addition of mild or moderate nsPEF may further improve therapeutic efficacy through the synergistic interaction demonstrated in this study. Second, the combination may enhance the effectiveness of nsPEF-based tumor ablation. nsPEF has been evaluated clinically for the treatment of skin [61] and liver tumors [44]; however, achieving complete ablation of large tumors remains a challenge. If suitable technologies can be developed for the coordinated delivery of both modalities, combining nsPEF with CAP may improve tumor control while potentially reducing the treatment intensity required from either modality alone. Future in vivo studies are needed to determine whether the synergistic effects observed in vitro can translate into improved therapeutic outcomes and expanded clinical applications.

## 4. Materials and Methods

### 4.1. Cell Culture

Pan02 murine pancreatic adenocarcinoma cells were originally provided by the Division of Cancer Treatment and Diagnosis (DCTD, NCI). 4T1 murine breast cancer cells were purchased from ATCC. Both cell lines were maintained in RPMI 1640 medium (ATCC 30-2001) supplemented with 10% fetal bovine serum and antibiotics (100 units/mL penicillin and 100 μg/mL streptomycin, Atlanta Biologicals, Oakwood, GA, USA). Frozen stocks (passage 4–6) were thawed for expansion, and cells with passage numbers 10 to 20 were used for all described experiments. Cells were tested periodically to ensure no mycoplasma contamination occurred.

### 4.2. In Vitro nsPEF and CAP Treatment

For nsPEF treatment, Pan02 or 4T1 cells (5 × 10^6^ cells/mL, 100 µL) in a 0.1 cm gap electroporation cuvette were exposed to 5 kV, 60 ns pulses at 1 Hz, with various pulse numbers (e.g., 20, 30, 35, and 100). To obtain a total volume of 300 µL per condition, three cuvettes were combined.

For cold atmospheric plasma (CAP) treatment, cells were seeded in 24-well plates (300 µL/well; 5 × 10^6^ cells/mL). Exposure was delivered using a nanosecond pulsed atmospheric pressure plasma jet (ns-APPJ; 200 ns, 9 kV, 2 kHz) as previously described [31]. Helium was used as the working gas at a fixed flow rate of 355 sccm. The high-voltage electrode nozzle tip was positioned at a fixed gap distance of 5 mm from the liquid surface. The CAP dosage was defined by exposure durations of 2, 3, 5, and 10 min (min).

For combination treatments, a 5 min interval was maintained between modalities. Two sequences were evaluated: (1) CAP (3 min) followed by nsPEF (20 pulses), and (2) nsPEF (20 pulses) followed by CAP (3 min). Untreated cells served as controls. A helium-gas-only control was not included based on prior validation showing negligible effects on Pan02 viability [31]. All conditions were tested in triplicate and on three separate dates.

### 4.3. Viability Determined by Metabolic Activity Assay

Following treatment, cell concentration was normalized to 1 × 10^6^ cells/mL. Suspensions (10 µL) were transferred to clear, flat-bottom 96-well plates containing 90 µL of complete medium and incubated for 18 h at 37 °C in a 5% CO_2_ humidified atmosphere. Cell viability was assessed using the WST-1 reagent (Roche Applied Science, Penzberg, Germany). WST-1 (10 µL) was added to each well, followed by a 2 h (h) incubation. Absorbance was measured at 450 nm (reference wavelength: 630 nm) using a MultiSkan MCC/340 microplate reader (Fisher Scientific, Waltham, MA, USA). Relative viability was calculated using the following formula: Treated sample (OD450-OD630)/control (OD450-OD630) × 100%. Pan02 cells without treatment but otherwise handled identically were used as the control.

For ROS scavenging experiments, cells were pre-incubated with 10 mM sodium pyruvate (Gibco, Waltham, MA, USA) for 30 min at 37 °C prior to nsPEF, CAP, or combination treatments.

### 4.4. Oxidative Stress and Mitochondrial Membrane Potential (ΔΨm) Analysis

Following the treatment, cells were incubated for 2 h at 37 °C. During the final 30 min, cells were stained with the following fluorescent probes: MitoSOX™ Red (5 µM; Invitrogen, Carlsbad, CA, USA) for mitochondrial superoxide, carboxy-H_2_DCFDA (5 µM; Invitrogen) for general intracellular ROS, and TMRE (100 nM; Invitrogen) for ΔΨm. Hoechst 33342 (Invitrogen) was used for nuclear counterstaining. Fluorescence was analyzed via flow cytometry using the following excitation/emission settings: MitoSOX (~510/580 nm), H_2_DCFDA (485/535 nm), and TMRE (~549/575 nm). Fluorescence microscopy was performed under identical staining conditions to visualize mitochondrial superoxide distribution. An initial FSC-A/SSC-A gate was applied to exclude debris and select the primary cell population. Singlet cells were then identified and gated to remove doublets and aggregates before ROS fluorescence analysis. The percentage of ROS-positive cells was subsequently determined from the gated singlet population using the appropriate fluorescence channel.

### 4.5. Caspase 3/7 Activity

Caspase activation was evaluated in the following groups: untreated control, nsPEF alone (30 or 100 pulses), CAP alone (5 or 10 min), and combination treatments (CAP 3 min + nsPEF 20 pulses; or nsPEF 20 pulses + CAP 3 min). Post-treatment, cells were plated (3 × 10^5^ cells/mL) in 6-well plates and incubated for 24 h. Caspase-3/7 activity was quantified using CellEvent™ Caspase-3/7 Green Detection Reagent (Invitrogen). The reagent (500 nM final concentration) was added to samples and incubated for 25 min at 37 °C in the dark. Green fluorescence (Ex/Em: 511/533 nm) was quantified by flow cytometry.

### 4.6. Western Blot Assay

Cells were harvested at indicated time points (2, 6, or 24 h) following treatment with nsPEF, CAP, combination treatment, or Staurosporine (STS; positive control). Lysates were prepared in RIPA buffer, and total protein was quantified via BCA assay. Proteins were resolved on 10% SDS–PAGE gels (160 V, 60 min) and transferred to PVDF membranes (60 V, 90 min). Membranes were blocked in Tris-buffered saline (TBS) for 30 min at room temperature and probed overnight at 4 °C with primary antibodies against cleaved caspase-3 and GAPDH (Cell Signaling Technology, Danvers, MA, USA). Bands were detected using goat anti-rabbit IRDye 680 secondary antibodies (LI-COR Biosciences, Lincoln, NE, USA) and visualized using a Bio-Rad ChemiDoc imaging system (Hercules, CA, USA).

### 4.7. Synergistic Quotient (SQ) Analysis

To evaluate whether the combined effects of nanosecond pulsed electric fields (nsPEF) and colatmospheric plasma (CAP) were additive, synergistic, or antagonistic, the synergistic quotient (SQ) was calculated as: SQ = AB/(A + B), where A and B represent the individual treatment effects of nsPEF and CAP, respectively, and AB represents the observed effect of the combined treatment. An SQ value > 1 indicates synergism, an SQ value = 1 indicates an additive effect, and an SQ value < 1 indicates antagonism. SQ values were calculated using percent cell death, ROS production, or other relevant biological endpoints as appropriate.

### 4.8. Statistical Analysis

Data are presented as mean ± standard deviation (SD) of at least three independent biological replicates performed on separate days to ensure reliability. Statistical significance was determined using one-way or two-way ANOVA, with *p* < 0.05 considered significant. Flow cytometry data were processed using FlowJo software (FlowJo V10). All statistical analyses and graphing were performed using GraphPad Prism 10.

## 5. Conclusions

The present study demonstrates that nanosecond pulsed electric fields (nsPEF) function as a potent bioelectric sensitizer for cold atmospheric plasma (CAP)-induced apoptosis in pancreatic cancer cells. This synergistic cytotoxicity is mediated by amplified mitochondrial ROS accumulation and the subsequent activation of the apoptotic cascade. Specifically, while nsPEF alone induces transient mitochondrial depolarization without triggering apoptotic death, it effectively primes cells to undergo robust ROS-dependent apoptosis when combined with CAP exposure. These findings provide a mechanistically grounded rationale for the development of nsPEF–CAP combination therapy as a novel, apoptosis-directed strategy for overcoming therapeutic resistance in refractory pancreatic cancer.

## Data Availability

The original contributions presented in the study are included in the article, further inquiries can be directed to the corresponding author.

## Abbreviations

The following abbreviations are used in this manuscript:

CAP/CP	Cold atomospheric plasma
Ctrl	Control
ΔΨm	Mitochondrial membrane potential
h	Hour
Hz/KHz	Hertz/kilohertz
kV/cm	Kilovolts per centimeter
µg	Microgram
mL	Milliliter
µL	Microliter
mM/µM	Millimole/micromole
min	Minute
ns	Nanosecond
nsPEF/NP	Nanosecond pulsed electric fields
SP	Sodium pyruvate
SQ	Synergistic quotient
